# Successful Treatment of Refractory Trichodynia With Onabotulinumtoxin-A

**DOI:** 10.7759/cureus.57009

**Published:** 2024-03-26

**Authors:** Faris A Alhomida, Sarah Alkhezzi, Rasha Alshammari, Bushra S Alasmari, Dalal A AlDosari, Monira AlNasser, Asem Almesfer, Saad AlSaadan

**Affiliations:** 1 Dermatology, King Fahad Medical City, Riyadh, SAU; 2 Dermatology, King Saud Bin Abdulaziz University for Health Sciences College of Medicine, Riyadh, SAU

**Keywords:** trichodynia, hair loss, botulinum toxin, botox, treatment, procedure, off-label, scalp dysesthesia, psychiatric co-morbidities, pain

## Abstract

Trichodynia is a common symptom, which is characterized by a painful, burning or stinging sensation of the scalp, often in patients presenting with hair loss. It is typically associated with co-morbid psychiatric conditions and remains challenging to treat, with no Food and Drug Administration (FDA) treatments currently available. We herein report the successful use of off-label onabotulinumtoxin-A in treating a patient with trichodynia who has failed conventional therapies.

## Introduction

Trichodynia is a common presenting symptom, characterized by a painful, burning or stinging sensation of the scalp that may be associated with an underlying trichological or co-morbid psychiatric disorder. The pain is typically reported to occur spontaneously, but activities such as hair combing, washing, or wearing hats are known triggers [1-5].

Although the exact etiology of trichodynia is yet to be known, it is postulated that it is likely multifactorial in origin. Substance P and other neuropeptides are thought to be released in response to a variety of stimuli, such as changes in temperature, emotional stimulus, hormonal factors, and mechanical or chemical stimulation. This, in turn, results in perifollicular inflammation from increased sensitization. Nutritional deficiencies such as iron, vitamin B12, vitamin D, and zinc have also been suggested to play a role, but the data remain inconclusive [2,3,6].

While the exact prevalence of trichodynia remains unknown, the literature reports that roughly one-third of patients presenting with hair loss may complain of features suggestive of trichodynia. Trichodynia has been reported to occur more frequently in women than men, with a reported female/male ratio of 2:1 [1,2].

Trichodynia can be both distressing for the patient and challenging for the clinician to treat, with no treatments currently available that are approved by the Food and Drug Administration (FDA) [5,7]. Furthermore, conventional therapies may predispose the patient to unwanted side effects [8]. Therefore, clinicians should be made aware of a potential novel treatment option that is not yet widely recognized as being efficacious for treating patients with trichodynia.

We herein report the successful use of off-label onabotulinumtoxin-A (BTX) in treating a patient with trichodynia who had failed conventional treatments. 

## Case presentation

A 52-year-old woman with refractory trichodynia presented to our tertiary dermatology clinic for further management of her condition. The patient reports a four-year history of trichodynia, with a pain numeric rating scale score (NRS) of 10/10 [9]. Her pain is maximally located in the regions of her occipital, vertex, and frontal scalp, in order of severity. The pain is triggered when brushing her hair or with any mechanical stimulation. She also endorses increased sensitivity to cold exposure. The pain has been significantly distressing for her. She also reports pruritus but is unsure if it is related to her pain. She denies any headaches associated with her pain. Her hair history is notable for having short hair and uses permanent hair color and over-the-counter hair-care products. She denies the use of any traction styling practices. The patient denies any habitual or compulsive hair-pulling. 

Her background medical history is notable for a remote history of right invasive ductal carcinoma status post-breast-conserving therapy, neoadjuvant chemotherapy, and radiotherapy. She is currently in remission and undergoes regular surveillance with her oncology team.

She also has a prior history of major depressive disorder (MDD) and insomnia. She has no known drug allergies. Previous treatments for her trichodynia included topical corticosteroids, topical 2% ketoconazole shampoo, and oral 25 mg hydroxyzine nightly, as needed, all of which failed to produce an effective treatment response. Interestingly, treatment of her co-morbid MDD with oral escitalopram and mirtazapine daily also failed to relieve her symptoms of trichodynia. She has since been off antidepressants. 

**Figure 1:**
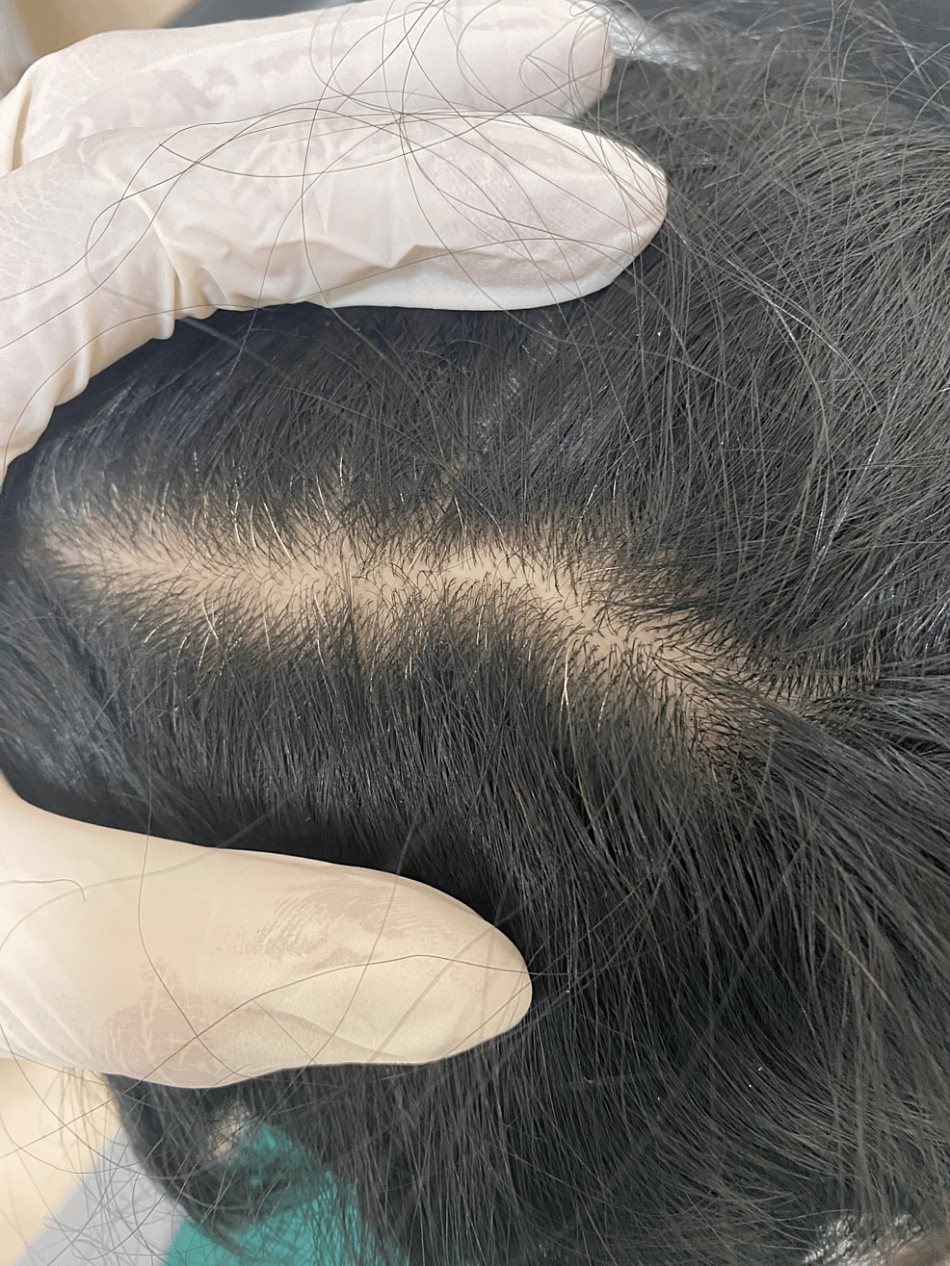
Cutaneous examination of the scalp revealing findings of normal hair density and hair diameter variation without evidence of erythema, scaling, or other gross pathologies.

Cutaneous examination of the scalp on presentation was normal with no obvious primary lesions identified (Figure 1). The hair-pull test was negative and there was no evidence of hair loss, scaling, erythema, or other gross pathologies. Dermoscopy findings were normal with no evident hair diameter variation, broken hairs, erythema, or scaling. Differential diagnoses considered included seborrheic dermatitis, trichotillomania, and trichodynia, which all could explain her pruritus and scalp pain. However, given no improvement with topical therapies or with antidepressant therapy and no gross pathologies found on clinical examination, the clinical diagnosis of trichodynia was confirmed, and the other differentials were ruled out. 

After discussing possible treatment options and collecting informed consent for the procedure, we elected to start the patient on a trial of a maximum of 100 units of BTX (BOTOX®, Allergan, Inc., Irvine, CA) diluted into 4 mL of normal saline 0.9% (NS) [10]. Pre-treatment topical lidocaine was applied to the patient’s scalp for roughly 30 minutes to an hour before the procedure. 0.1 mL aliquot (2.5 units of BTX) of the prepared BTX solution was injected subcutaneously throughout the scalp, distributed in a 1-3 cm grid pattern, emphasizing the area of maximal point tenderness along her occipital, vertex, and frontal scalp. The number of injection points was not counted or limited. The patient experienced mild injection-site pain during the procedure but otherwise tolerated the procedure well with no other reported side effects.

At one-month follow-up, the patient reported her symptoms had significantly improved and was asymptomatic with an NRS of 0. By six-week post-treatment with BTX, the patient reported noticing her symptoms gradually returning, with an NRS of 6 by the second month. Finally, by three-month follow-up, she reported her symptoms had returned as they were at baseline but the pain occasionally waxes and wanes. Thus, we elected to continue with another cycle with BTX.

By one-year follow-up, she reports significant improvement with BTX. Given the waxing and waning nature of her condition, the regular cycles of BTX helped prolong her symptom-free period for nearly six months. She continues to follow up with dermatology every 3-5 months for BTX treatments of her trichodynia. 

## Discussion

Although trichodynia is a common presentation, it remains difficult to treat, with no FDA-approved treatments available. Conventional treatments proposed in the literature include topical therapies such as shampoos, corticosteroids, calcineurin inhibitors, and capsaicin and topical anesthetics. Systemic therapies suggested include oral L-cysteine preparations, antihistamines, systemic corticosteroids, and beta-blockers such as propranolol [2,4,11].

According to the literature, roughly 76% of patients with trichodynia may have co-morbid psychiatric conditions [4]. Given this association, various psychotropic medications have been reported to be of some use. These include gabapentin and pregabalin and antidepressants such as tricyclic antidepressants, selective serotonin reuptake inhibitors, and selective serotonin-norepinephrine reuptake inhibitors [2,4,8]. Despite the commonly reported use of antidepressants for the management of trichodynia, our patient failed to achieve an effective treatment response.

Off-label BTX has been shown in multiple, randomized, placebo-controlled studies to be an effective treatment modality in the management of various neuropathic disorders including plantar fasciitis, focal painful neuropathies, mechanical allodynia, and myofascial pain [12-14]. It has been postulated that BTX may directly inhibit neurogenic inflammation via an inhibitory effect on the release of various neurotransmitters from afferent nerve fibers responsible for eliciting the pain sensation including substance-P, calcitonin gene-related peptide, and glutamate [5,7,12-14]. The exact mechanism of action of BTX in diminishing the pain response is yet to be fully understood.

Our findings are consistent with previous reports of the successful use of off-label BTX as a potential treatment modality for patients struggling with trichodynia [5,7]. Trimboli et al. pioneered the first use of BTX for the treatment of trichodynia and relied on a dose of 200 units. This was distributed along 40 injection points on the scalp, with each point receiving 0.1 mL (5 units of BTX) of the prepared solution. They reported significant patient improvement, with a reduction of the NRS from 8 to 6 by three-month follow-up. They repeated the procedure at this time, and by six-month follow-up, the patient was asymptomatic with an NRS of 0. The patient remained asymptomatic by nine-month follow-up. The patient received three cycles of BTX in total, one every three months. This is similar to our findings, which suggest that BTX efficacy tends to wear off gradually. Furthermore, these findings, taken together, illustrate that BTX may have a prolonged effect with repeated courses when scheduled roughly every three months [5].

Similarly, Phan et al. reported a placebo-controlled, split-scalp trial on a 59-year-old man with 40 units of BTX diluted in 1 mL of NS (2 units of BTX per 0.05 mL of NS). This was distributed along 20 injection points in a 3 cm grid pattern on one half of the scalp as compared to placebo on the other side. They found considerable improvements in the patient’s pain symptoms at the eight-week follow-up and a greater reduction of pain scores on the treated side than the placebo. Comparably, their findings also suggest that the pain-relieving effect of BTX seemed to diminish by the 16-week follow-up [7].

For our patient, we relied on the standard dilution of BTX used in the management of axillary hyperhidrosis. This, in part, is due to the established safety of the drug at this dilution. The standard dilution is typically reported as 100 units per 4 mL of NS or 2.5 units of BTX per 0.1 mL [10]. We find that this dilution provides the clinician the convenience of not having to remember a new dilution formula, while still providing safe and effective pain relief for the patient. Our findings suggest that these BTX cycles may need to be repeated every 3-5 months, depending on the patient. 

However, more research is warranted to confirm the exact dosage needed and confirm the mechanism of action, duration of sustained treatment response, and safety of BTX in managing patients presenting with trichodynia. Additionally, it would be interesting to see if the duration of pain relief can be further extended with zinc supplementation. Prior reports have suggested that when BTX was used for other indications, supplementing with oral 50 mg of zinc daily for four days prior to the procedure resulted in a roughly 30% increase in the toxin’s effect and duration [15,16]. Thus, larger clinical trials are needed to address these clinical concerns.

## Conclusions

In conclusion, trichodynia, despite being a commonly encountered problem, remains challenging to treat and distressing to patients. Hence, a novel management approach is warranted, particularly for patients who have failed other measures. The off-label use of BTX has proven to be effective in managing our patient and may be a promising treatment modality that other clinicians can rely on, for their patients with trichodynia. A multidisciplinary treatment approach, however, is still recommended to better achieve reliable patient outcomes.
